# Telemedicine in Intensive Care Units: Scoping Review

**DOI:** 10.2196/32264

**Published:** 2021-11-03

**Authors:** Camille Guinemer, Martin Boeker, Daniel Fürstenau, Akira-Sebastian Poncette, Björn Weiss, Rudolf Mörgeli, Felix Balzer

**Affiliations:** 1 Institute of Medical Informatics Charité-Universitätsmedizin Berlin, Corporate member of Freie Universität Berlin Humboldt-Universität zu Berlin, and Berlin Institute of Health Berlin Germany; 2 Intelligence and Informatics in Medicine, Medical Center rechts der Isar School of Medicine Technical University of Munich Munich Germany; 3 Department of Digitalization Copenhagen Business School Copenhagen Denmark; 4 Department of Anesthesiology and Operative Intensive Care Medicine (CCM, CVK) Charité-Universitätsmedizin Berlin, Corporate member of Freie Universität Berlin Humboldt-Universität zu Berlin, and Berlin Institute of Health Berlin Germany

**Keywords:** tele-ICU, telemedicine, critical care, implementation, telehealth, health care system, intensive care unit, health technology, digital health, care compliance, tertiary hospitals, hospital, review

## Abstract

**Background:**

The role of telemedicine in intensive care has been increasing steadily. Tele–intensive care unit (ICU) interventions are varied and can be used in different levels of treatment, often with direct implications for the intensive care processes. Although a substantial body of primary and secondary literature has been published on the topic, there is a need for broadening the understanding of the organizational factors influencing the effectiveness of telemedical interventions in the ICU.

**Objective:**

This scoping review aims to provide a map of existing evidence on tele-ICU interventions, focusing on the analysis of the implementation context and identifying areas for further technological research.

**Methods:**

A research protocol outlining the method has been published in JMIR Research Protocols. This review follows the PRISMA-ScR (Preferred Reporting Items for Systematic Reviews and Meta-Analyses Extension for Scoping Reviews). A core research team was assembled to provide feedback and discuss findings.

**Results:**

A total of 3019 results were retrieved. After screening, 25 studies were included in the final analysis. We were able to characterize the context of tele-ICU studies and identify three use cases for tele-ICU interventions. The first use case is *extending coverage*, which describes interventions aimed at extending the availability of intensive care capabilities. The second use case is *improving compliance*, which includes interventions targeted at improving patient safety, intensive care best practices, and quality of care. The third use case, *facilitating transfer*, describes telemedicine interventions targeted toward the management of patient transfers to or from the ICU.

**Conclusions:**

The benefits of tele-ICU interventions have been well documented for centralized systems aimed at extending critical care capabilities in a community setting and improving care compliance in tertiary hospitals. No strong evidence has been found on the reduction of patient transfers following tele-ICU intervention.

**International Registered Report Identifier (IRRID):**

RR2-10.2196/19695

## Introduction

Telemedicine has been increasingly used in intensive care, and approximately 15% of intensive care beds in the United States currently partake in telemedical programs [1-3]. A range of rationales for the implementation of telemedical systems in intensive care has been suggested. Tele–intensive care unit (ICU) technologies have been used to address staffing shortage in intensive care and as a cost-effective response not only to a lack of intensive care availability in some areas but also as a means of increasing adherence to evidence-based best practices using benchmark performance data [3-5].

The American Telemedicine Association defines tele-ICU as “a network of audiovisual communication and computer systems that provide the foundation for a collaborative, interprofessional care model focusing on critically ill patients” [3]. Tele-ICU interventions are varied, can be offered in different levels of intensive care service, and can be customized to meet the specific intensive care needs of hospitals [3,5-7]. For example, some tele-ICU systems provide 24/7 remote monitoring staffed by intensivists, while other systems provide scheduled remote intensivist consultations during nighttime only.

The main characteristics of tele-ICU systems have been well described in the literature. First, technical architectures can be described as centralized or decentralized. Centralized architecture features a command center, or a *cockpit*, connecting one or multiple centers. Decentralized systems (also named *virtual consultant*) allow one-on-one connections without the need for central coordination [3]. Second, staff allocation and availability can vary (eg, day presence or 24/7) [8]. Third, the mode of interaction between telemedicine teams and bedside staff may allow various levels of staff reactivity (reactive vs proactive to patient alerts) and intervention scope (minimal intervention allowed vs full discretion on patient care) [4]. Several guidelines, such as the US [3] or the German Guidelines for Telemedicine in Intensive Medicine [9], provide general recommendations on aspects of equipment, staffing, and organization for implementing tele-ICU systems.

A significant body of primary and secondary literature has been published on ICU telemedical interventions [10]. To date, 9 systematic reviews and 9 other review types have been published on this topic [11], as well as 3 meta-analyses with a focus on medical outcomes (eg, hospital mortality and length of stay) [12]. In previous reviews, the results of tele-ICU interventions have been characterized as heterogeneous [13,14]. Although positive medical outcomes could be detected in some interventions, other contexts could only demonstrate mixed or no positive results at all [4,14,15]. Authors have suggested that the context of implementation may be a factor in explaining the variability of these results. We define context of implementation as the clinical structures and processes where telemedical interventions are deployed [16]. It has been suggested that the efficacy of tele-ICU interventions is dependent on where and how they are deployed in the organization [6,10], and there is a need for broadening the understanding of the organizational factors influencing the efficacy of tele-ICU interventions [8]. We found that no previous study has attempted to provide a review of current evidence by systematically analyzing the implementation setup and context.

This scoping review seeks to address a research gap on the characterization of the context of implementation for tele-ICU interventions [14,17]. The first objective is to characterize the implementation context of tele-ICU interventions with a consistent set of domains on hospital organization. The second objective is to characterize the configurations and structures of tele-ICU systems in relation to their context of implementation. The third objective is to describe the outcomes of tele-ICU interventions and to characterize current evidence according to their intervention contexts.

## Methods

A research protocol for this review was published in JMIR Research Protocols in December 2020 [11], which was developed in accordance with the PRISMA-ScR (Preferred Reporting Items for Systematic Reviews and Meta-Analyses Extension for Scoping Reviews) and best practices advanced by Arksey and O’Malley [18] and the Joanna Briggs Institute [19]. The method included the steps *identification of relevant studies*, *selection of study*, *data charting*, and *data collating*.

For the step *identification of relevant studies*, a search for peer-reviewed studies in the databases Web of Science Core Collection, MEDLINE, ERIC, PsycINFO, PSYNDEX, CINAHL, and IEEE was performed without date restrictions. Manual searches were performed additionally to identify gray literature. The search query was developed according to the guidelines of the Peer Review of Electronic Search Strategies and included keywords on the topics of intensive care and telemedicine. The full queries are provided in Multimedia Appendix 1. The search records were downloaded in the reference software Citavi version 6 (Swiss Academic Software).

In the step *selection of study*, both titles and abstracts were screened, and studies not dealing with a relevant topic or method were removed. Results were then screened to find articles where the PICO (Patient, Intervention, Comparison, Outcome) framework could be identified. We included articles with at least three of the PICO criteria summarized in Textbox 1. Studies concerning interventions in neonatal and pediatric ICUs were excluded from this scoping review.

In the step *data charting*, article information was collected and classified into extraction sheets according to the five domains defined in the review protocol (see Textbox 2).

In the step *data collating, summarizing, and reporting*, the information was organized and clustered into an evidence map. The evidence map provided a summary of the scoping review results. During the review process, a core research team was created to provide feedback and discuss findings. The research team was composed of a doctoral researcher with a background in health economics (author CG), a medical data science professor (author FB), a medical informatics professor (author MB), an anesthesiologist with intensive care specialty and main coordinator of a tele-ICU project (author BW), an anesthesiology researcher with a specialty in digital health (author ASP), a professor of digitalization (author DF), and an anesthesiologist with intensive care specialty (author RM). The research team was asked to consider the information from data charting, provide insights, and discuss results. Differing views were resolved through discussion until consensus was reached.

**PICO (Patients, Intervention, Comparison, Outcomes) criteria.**

**Patient**

Participants provided telemedical intensive care.

**Intervention**

Telemedical system implemented with one more an intensive care units (ICUs).

**Comparison**

Comparison with the standard of care without tele-ICU intervention.

**Outcomes**

All outcomes eligible for inclusion.

**Data charting domains.**

**Implementation context**

*A. Clinical focus*

Level of intensive care specialization. Generalist (medical intensive care unit [ICU], surgical ICU) or specialized clinical focus (ie, sepsis, cardiology, neurocritical).

*B. ICU type*

Level of intensivist involvement in patient care. Defined by staffing model of ICU (ie, open vs closed ICU models).

*C. Hospital type*

Category of hospital involved in tele-ICU intervention (ie, tertiary or community hospital). Community hospitals are defined as nonfederal, short-term general hospitals under 500 beds [20].

*D. System configuration*

Technical architecture (ie, centralized vs decentralized), staff allocation (ie, continuous vs scheduled), and mode of communication of the tele-ICU system (ie, high or low data intensity).

*E. Implementation rationale*

Main rationale provided in the study for tele-ICU intervention, use case for telemedical system in the ICU.

## Results

### Selection of Relevant Studies

**Figure 1:**
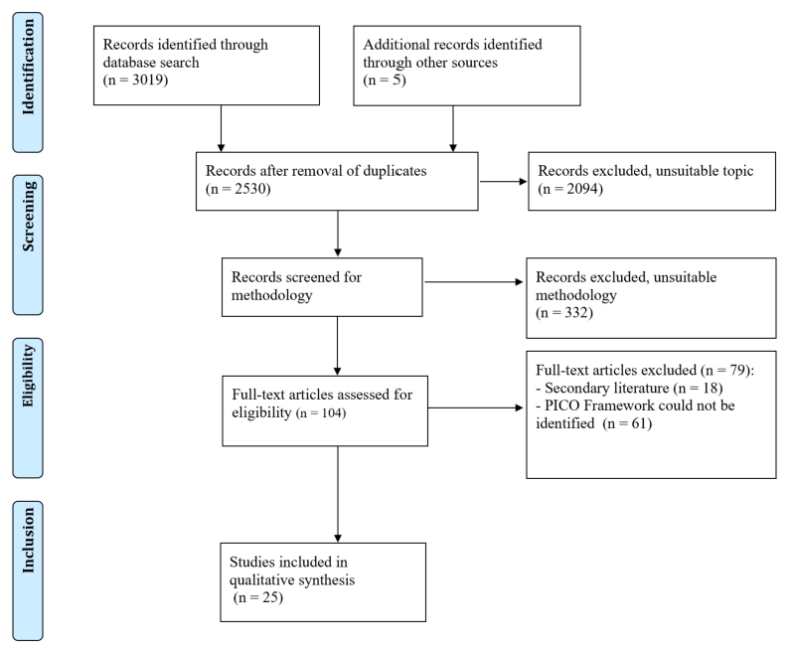
PRISMA (Preferred Reporting Items for Systematic Reviews and Meta-Analyses) flowchart. PICO: Patient, Intervention, Comparison, Outcomes.

The flowchart in Figure 1 outlines the records yielded by the search. A total of 3019 results were retrieved, including 489 duplicates. After screening, 104 records were eligible for full-text analysis and 25 were included in the final analysis.

### Characteristics of Tele-ICU Studies Included in the Scoping Review

The 25 studies included in this review were published between 2004 and 2019. Out of 25 articles, 21 (84%) referred to tele-ICU implementation within the Unites States, while the remaining papers described implementation in Germany, India, Australia, and Saudi Arabia. Regarding the research methods used in the studies, we found that 21 articles used pre-post comparison designs, of which only 7 included a control group. The pre-post design has been described as a quasi-experimental research design [12,21], for which a random assignment of patients between treatment and control group was not performed. The remaining 4 publications used other methods, such as interrupted time series, and half of these included a control group. We found no examples of randomized controlled trials.

### Results From Data Charting

Table 1 summarizes the data charting results for the 5 research domains and provides definitions for each category.

First, we outline results for the domains pertaining to context of implementation (domains A to C). For domain A, most telemedical implementations did not have a specific clinical focus (n=21, 84% of the studies), with only a few cases of specialized interventions. For domain B, tele-ICU interventions were predominantly implemented in ICUs featuring aspects of the open model. In these interventions, the primary physicians or surgeons retained full responsibility for the patient (n=10, 40% of the studies) or with limited intensivist involvement only (n=9, 36% of cases open/closed). Regarding domain C, although 44% (n=11 studies) of interventions were implemented in tertiary hospitals, a large subset was in community settings and in organizations spanning both tertiary and community hospital settings.

**Figure 2:**
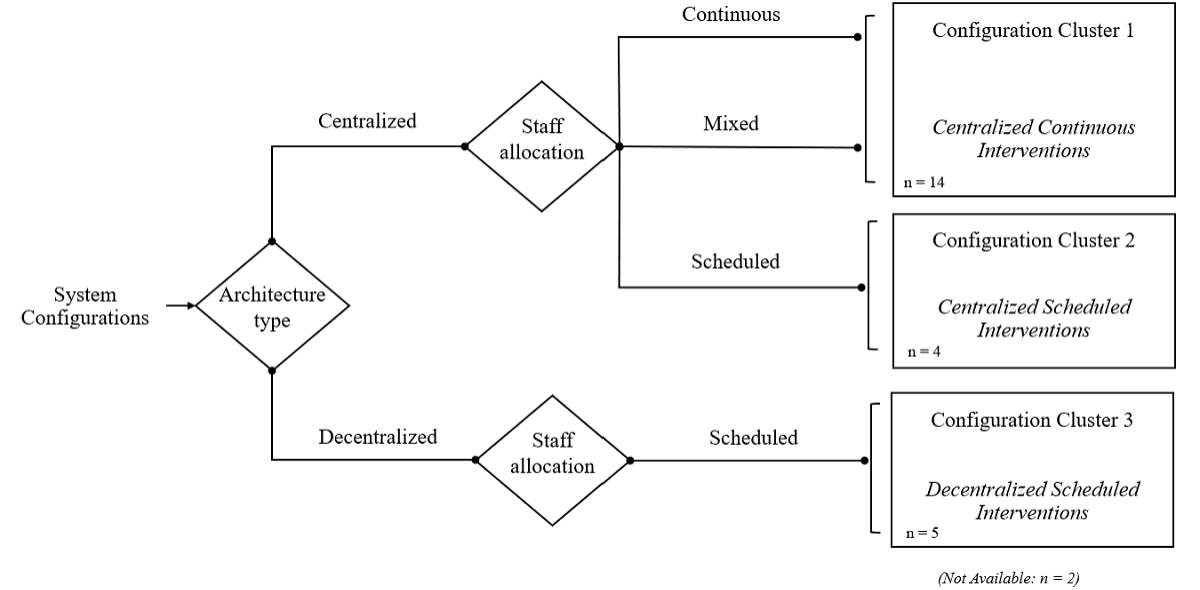
Clustering system configurations.

Second, concerning the system configuration results in domain D, centralized architectures (eg, tele-ICU Command Center) were the predominant implementation setup. Relating to the staffing model, the continuous care setup was used in 13 (52%) of the studies, where the remote care team assumes constant patient monitoring. Scheduled interventions (eg, daily intensive care rounds) were found in 9 (36%) cases. Finally, most telemedical systems (n=19, 76%) enabled remote real-time access to patient data. To summarize this information, we classified the system configurations into three clusters, as outlined in Figure 2.

Finally, concerning the implementation rationale defined in domain E, three main use cases were defined for tele-ICU interventions. We classified 13 (52%) publications under the use case 1 summarized by the term *extending coverage*. In this group, studies cited intensivist shortage, need for additional intensivist coverage, and extension of intensivist resources as a rationale for the intervention. A total of 10 (40%) studies were classified under use case 2, summarized by the term *improving compliance*. In this group, studies cited the increase in adherence to compliance with care bundles, clinical practice guidelines, or care quality initiatives as the main rationale. We classified two studies in use case 3, summarized by the term *facilitating transfer*. Studies in this category cited the screening or monitoring of patients prior to transfer to or from an ICU as the main rationale.

**Table 1:** Data charting results: interventions and context.

Domain and category					Definition			Studies (N=25), n (%)
**Implementation context**								
	**Clinical focus**							
		General	No specific clinical focus identified (MICU^a^, SICU^b^)			21 (84)		
		Specialized	Specific clinical focus (ie, sepsis, cardiology, neurocritical)			4 (16)		
	**ICU^c^ type**							
		Open	Primary physician has full-time responsibility for patient care			10 (40)		
		Open/closed	Features of both open and closed models			9 (36)		
		Closed	Intensivists available with full responsibility for patient care			6 (24)		
	**Hospital type**							
		Tertiary	Tertiary care institutions or teaching hospitals			11 (44)		
		Mixed	Care organization spanning tertiary and community settings			4 (16)		
		Community	Community hospitals or small medical facility			9 (36)		
		Not available	N/A^d^			1 (4)		
**System configuration**								
	Continuous			Continuous patient critical care monitoring			5 (20)	
	Mixed			Continuous monitoring including scheduled rounds			9 (36)	
	Scheduled			Scheduled consultation at regular interval. Virtual rounds.			9 (36)	
	Not available			Insufficient information provided			2 (8)	
	Centralized			Tele-ICU Command Center or Hub centralizing patient care			19 (76)	
	Decentralized			Distributed architecture without centralized hub			5 (20)	
	Not available			N/A			1 (4)	
	Direct access			Direct staff remote access to patient data			18 (72)	
	Limited access			Limited staff remote access (screen sharing) to patient data			4 (16)	
	Not available			N/A			3 (12)	
**Implementation rationale**								
	Coverage			Intensivist shortage, provision of extended coverage			13 (52)	
	Compliance			Adherence and compliance to critical care guidelines			10 (40)	
	Transfer			Patients screening or triage for transfers to or from ICU			2 (8)	

^a^MICU: medical intensive care unit.

^b^SICU: surgical intensive care unit.

^c^ICU: intensive care unit.

^d^N/A: not applicable.

### Intervention Outcomes

This section presents results on the range of outcomes that were found in the studies on ICU implementation, which are summarized in Table 2.

**Figure 3:**
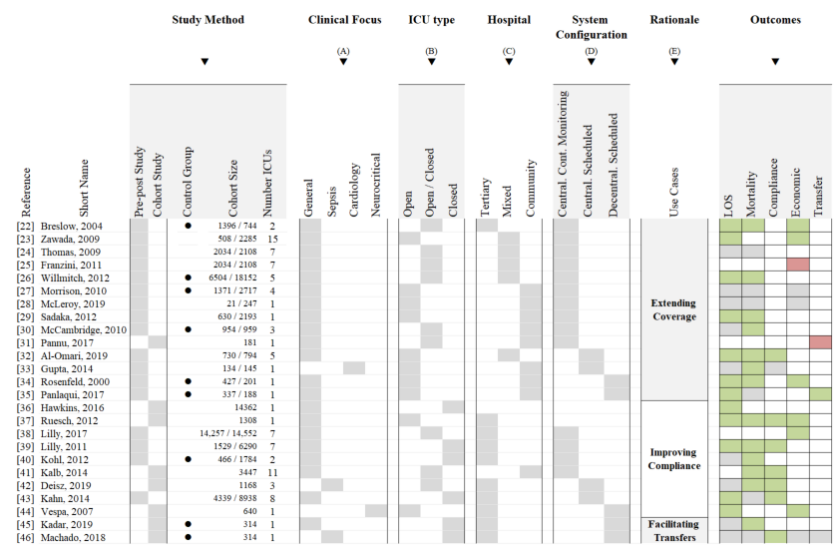
Evidence map [22-46]. ICU: intensive care unit; LOS: length of stay.

First, a significant subset of studies provided results on at least one medical outcome. Effect of tele-ICU intervention on length of stay (LOS) was reported in 21 (84%) studies. This outcome was defined as the number of inpatient days for the episode of care in the ICU or in aggregate in the hospital. Results on mortality rates were provided in 19 (76%) studies, including ICU and hospital mortality. In 12 studies, reduction in LOS was found to be significant. Reduction in mortality was significant in 13 studies. Second, 8 (32%) studies measured the rate of adherence to best practices and guidelines implementation, summarized by the term *compliance*. A large subset indicated a statistically significant increase in the level of adherence to ICU standards. Third, under the header *economics,* 9 (36%) studies provided results regarding cost-effectiveness of tele-ICU interventions. In this subset, 6 (67%) studies reported interventions as being cost-effective. Lastly, 2 studies in the category *transfer* measured changes in rate of patient transfer following intervention. One study measured the number of transfers within the same facility (eg, for preadmission diagnostic) and another the number of transfers to another facility (eg, for advanced care). Finally, we note that none of the studies included patient satisfaction scores. These results are summarized as an evidence map in Figure 3.

**Table 2:** Data charting results: outcomes.

Outcome category	Reporting on outcome, n	Of which reporting positive results, n
Length of stay	21	12
Mortality	19	13
Compliance	8	7
Economics	9	6
Transfer	2	1

## Discussion

### Principal Findings

This scoping review provided an overview of the literature on telemedical interventions in the ICU. Based on a set of defined domains, we were able to characterize the context of tele-ICU studies and identify three use cases for tele-ICU interventions. This analysis aimed to identify common features within the heterogenous use of telemedical systems. Recent research findings relevant for implementation under each use case were outlined.

The first use case, *extending coverage*, included interventions aimed at increasing intensive care coverage in contexts where it is not (or only partially) available at the bedside. This use case was found predominantly in community hospitals having limited onsite critical care capacity. The second use case, *improving compliance*, included interventions targeted at improving patient safety, intensive care best practices and quality of care. These interventions were found primarily in tertiary care context. The third use case, *facilitating transfer*, included telemedicine interventions targeting toward the management of patient transfers to or from the ICU.

### Use Case: Extending Coverage

Interventions were predominantly found in community hospitals and in mixed community/tertiary contexts (eg, hospital groups spanning one or several community branches). Tele-ICU systems in this use case have been used to address specific issues related to the delivery of critical care in community and rural areas. Particularly in the United States, recent surveys indicate that hospitalists (ie, physicians whose main focus is on general medical care of patients who are hospitalized [47]) are still the main physician in rural and community settings, reflecting a general shortage in intensive care staffing [48]. Although community hospitals face difficulties in hiring qualified critical care personnel, some of them are subject to minimum requirements to have full intensivist staffing during the day [49,50]. In underserved areas, tele-ICU implementation can therefore represent a valuable solution for the onsite provision of intensive care expertise [51].

The predominant tele-ICU system configuration in this use case was a centralized system featuring continuous remote staff intervention from a workstation, with direct involvement in patient care (configuration cluster: *centralized continuous monitoring*). Team cooperation and sharing of responsibility over patient care between the bedside and remote team are central issues in this type of configuration. Our analysis showed that different modalities of a remote care team have been implemented. In some interventions, the main role of the remote team was to consult and advise the bedside team (Zawada et al [23], McLeroy et al [28], and Al-Omari et al [32]; n=3, 12% of studies), whereas in other cases, remote staff were granted a different level of authority on patient care at the discretion of the bedside team (Sadaka et al [29], Morrison et al [27], Thomas [24], Willmitch et al [26], Franzini et al [25], and Breslow et al [22]; n=6, 24% of studies). Achieving an appropriate degree of cooperation between bedside and remote care has been described as a success factor of telemedical interventions [10,52]. Recent literature on the impact of tele-ICU interventions suggest that effectiveness is enhanced when comanagement and clear autonomy of the remote care team are allowed [10,36]. Particularly for intensive care nurses, there is a need to establish clear rules of engagement to avoid conflicting orders between bedside and remote teams [53]. A recent ethnographic review also indicates that the perceived value of the intervention by bedside staff is a contributing factor to the success of the intervention [14]. The core research group discussed in particular the aspect of bedside physician’s trust in the remote specialist. As an example, situations where an experienced physician of a nonacademic hospital in a rural area collaborates with a less experienced physician at a university hospital telemedical center can raise the issues of perceived value and trust between remote and bedside personnel. Therefore, the involvement of bedside staff during planning, system implementation, and training is recommended to enhance organizational acceptance [54,55]. As part of the implementation process, actions targeted at team cohesion (eg, team building) and use of standardized communication practices between teams can enhance the implementation of new workflows [56,57]. Implementation of health technology can lead to changes in work practice inside the care team, in particular for nursing and support staff [58]. Clear definition of the roles, responsibility, and composition of the team should therefore be addressed early on during the planning of the intervention.

Implementation of tele-ICU systems has been advanced as a solution for community hospitals facing the challenge of sustaining the cost of maintaining a local ICU with high standard of care. Economic evaluations of tele-ICU interventions are therefore an important aspect for consideration in the community settings. With tele-ICU systems, community hospitals have the potential to treat patients with a higher case mix index locally and at lower cost [51]. At the same time, high cost of tele-ICU systems has been described as a barrier to implementation [59]. Our finding indicates that studies on cost-effectiveness in this use case have not yielded uniform results. The included studies in this review have used heterogeneous approaches to estimate savings and revenue increase following tele-ICU implementation. We corroborate previous observations concerning the lack of transparency and comprehensive data on costs, which hinder comparisons and clear statements regarding cost-efficacy [59,60].

### Use Case: Improving Compliance

In this use case, ICU systems were primarily configured as scheduled daily rounds from a tele-ICU center (configuration cluster *centralized scheduled interventions*; n=4, 16% of studies) and decentralized systems allowing expert remote consultations (configuration cluster *decentralized scheduled interventions*; n=5, 20% of studies). Interventions in the use case are mainly focused on advancing adherence to best practices in the ICU and increasing patient safety. They consisted in establishing critical care processes in which the remote care teams monitor relevant quality indicators (eg, prophylaxis for stress ulcer, ventilator-associated pneumonia, or deep vein thrombosis). In our analysis, there is some evidence that ICU interventions are conducive to higher adherence to best practices in the ICU and enhance patient safety, thereby corroborating earlier observations on efficacy [51]. We found that most evidence for this type of intervention has been reported in tertiary care hospitals with a closed or mixed ICU model. Additional research would be needed to understand how this type of intervention could be beneficial in other hospital contexts. The review highlighted an intervention specialized on prevention of sepsis (Deisz et al [42]), for which compliance to the care bundle was found to remain low [61,62].

We hypothesize that the efficacy of these interventions is derived from a combination of change in the care process (eg, increased use of reminders and checklists) and the use of decision support systems (eg, smart alerts). Tele-ICU systems are conducive to real-time benchmarking of performance and allow targeted actions to enhance compliance and care quality. Surveillance systems can improve resource allocation by allowing for more rapid response time and faster escalation of the most acute cases [54]. Additionally, tele-ICU systems have been shown to reduce alarm fatigue through triage and curation of automatic alerts by remote care teams [51,59]. In recent literature, the potential of population management systems allowing targeted interventions on patients with high risk factors has been highlighted [63]. Significant amount of data generated by tele-ICU systems can be leveraged for the development of advanced applications [64]. A recent systematic review on telemedicine with clinical decision support for critical care indicated the need for further research on the use and efficacy of advanced applications in units equipped with telemedical systems [65].

### Use Case: Facilitating Transfer

Interventions in this use case are aimed at supporting patient transfers between hospitals (ie, referral to higher level hospital) and monitoring patients during admission in the ICU from another department (eg, emergency department). This form of intervention has been described in the literature as consultative critical care services [66]. One study in the review documented the benefit of these interventions for patients in the emergency department with suspected sepsis diagnosis [54]. Based on the studies in the review, we can corroborate previous reports that no strong evidence has been found regarding the benefit on the number of transfers for this type of interventions [67].

### Limitations

Our approach has multiple limitations. First, the studies included in the review used heterogeneous research methods. Authors provided varying degree of details to describe the intervention setup and implementation context. Aspects such as staff interaction and level of autonomy have been provided only in a limited number of studies, so that our ability to draw generalizable conclusions on these aspects of tele-ICU interventions has been limited. Second, relying on the expertise of the core research group to complete the data charting was qualitative in nature and potentially subject to bias. A discussion process section was established to mitigate the interpretation bias in our approach. Third, the scope of this review was limited to the implementation of tele-ICU systems for adult patients, and critical care telemedical interventions have also been documented in pediatrics and neonatology. Some of our conclusions might therefore not be applicable to these settings.

### Conclusion

Tele-ICU systems have been deployed in numerous implementation contexts, which we characterized in three main use cases. The benefits of tele-ICU interventions have been well documented for centralized systems aimed at extending critical care capabilities in community settings and improving care compliance in tertiary hospitals. This scoping review provides teams involved in the implementation of tele-ICU systems with an overview of existing evidence on the technology. It highlights factors that are conducive to successful implementation for different critical care context. This review also mentions areas for future research on tele-ICU interventions. Furthermore, the framework for describing the implementation context used in this scoping review could be used for analyzing other types of telemedical interventions or other domains of intervention (eg, traumatology, pediatrics, neonatology).

## Appendix

Multimedia Appendix 1 Search queries for peer-reviewed studies in literature databases.

## Abbreviations

ICU: intensive care unit
PICO: Patient, Intervention, Comparison, Outcome
PRISMA-ScR: Preferred Reporting Items for Systematic Reviews and Meta-Analyses Extension for Scoping Reviews
