# Supplementary material for: Telemedicine in Intensive Care Units: Scoping Review
Source: J Med Internet Res. 2021 Nov 3;23(11):e32264. doi: 10.2196/32264 (PMC8600441; doi:10.2196/32264)
Supplement: Multimedia Appendix 1 [file jmir_v23i11e32264_app1.pdf]

Multimedia Appendix 1.
Search queries for peer-reviewed studies in literature databases

Search Engine	Query Text
Web of Science Search	TOPIC: (<br><br>("intensive care unit" OR<br>icu OR<br>“intensive care” OR<br>“acute care” OR<br>"critical care")<br><br>AND<br><br>(tele-ICU OR<br>teleICU OR<br>“tele ICU” OR<br>"Digital health" OR<br>e-health OR<br>telemedicine OR<br>telecare OR<br>telehealth OR<br>ehealth OR<br>mhealth OR<br>“remote presence” OR<br>“Virtual ICU”<br>"Digital Intervention")<br><br>)
EBSCO Host	("intensive care unit" OR<br>icu OR<br>“acute care” OR<br>"critical care")<br><br>AND<br><br>(tele-ICU OR<br>teleICU OR<br>“tele ICU” OR<br>"Digital health" OR<br>e-health OR<br>telemedicine OR<br>telecare OR<br>telehealth OR<br>ehealth OR<br>mhealth OR<br>"Digital Intervention")<br><br>)
IEEE Xplore	("intensive care unit" OR<br>icu OR<br>“acute care” OR<br>"critical care")

MULTIMEDIA APPENDIX 1.
Search queries for peer-reviewed studies in literature databases

AND

(tele-ICU OR
teleICU OR
“tele ICU” OR
"Digital health" OR
e-health OR
telemedicine OR
telecare OR
telehealth OR
ehealth OR
mhealth OR
"Digital Intervention")

)
